# Usual care including home exercise with versus without spa therapy for chronic low back pain: protocol for the LOMBATHERM’ study, a multicentric randomised controlled trial

**DOI:** 10.1186/s13063-020-04271-9

**Published:** 2020-05-11

**Authors:** Romain Forestier, Carey Suehs, Alain Françon, Marc Marty, Stéphane Genevay, Jérémie Sellam, Claire Chauveton, Fatma Begüm Erol Forestier, Nicolas Molinari

**Affiliations:** 1 Centre de Recherche Rhumatologique et Thermal, 15 avenue Charles de Gaulle, 73100 Aix-les-Bains, France; 2 Departments of Medical Information and Respiratory Diseases, Univ Montpellier, CHU Montpellier, Montpellier, France; 3 Department of Rheumatology, APHP – Hôpital Henri Mondor, Créteil, France; 4 Department of Rheumatology, Geneva University Hospitals, Geneva, Switzerland; 5 Department of Rheumatology, APHP – Hôpital Saint-Antoine, Sorbonne Université, Inserm URMS_938, Paris, France; 6 Delegation for Clinical Research and Innovation, Univ Montpellier, CHU Montpellier, Montpellier, France; 7 IMAG, CNRS, Univ Montpellier, CHU Montpellier, Montpellier, France

**Keywords:** Crenobalneotherapy, Underwater massages, Mud application, Water exercises, Low back pain, Spa therapy

## Abstract

**Background:**

Low back pain is highly prevalent and a major source of disability worldwide. Spa therapy is frequently used to treat low back pain, but the associated level of evidence for efficacy is insufficient. To fill this knowledge gap, this protocol proposes an appropriately powered, prospective, evaluator-blinded, multi-centre, two-parallel-arm, randomised (1:1), controlled trial that will compare spa therapy in addition to usual care including home exercise (UCHE) versus UCHE alone for the treatment of chronic low back pain.

**Methods:**

Eligible patients (anticipated sample size of 358) will have had low back pain for more than 3 months and scores for pain greater than 40 mm on a visual analogue scale (VAS). Following initial consent for UCHE and baseline evaluations, patients are randomised (1:1) to UCHE alone, or UCHE plus spa therapy (18 days of mud packs, underwater massages, showers and water exercises under medical supervision). Patients in the latter arm will be requested to sign an additional consent form as per Zelen randomisation. Follow-up visits will occur at approximately months 1, 6 and 12 and (along with baseline assessments) will cover changes over time in VAS pain scores, the impact of lower back pain on daily life (the Rolland and Morris Disability Questionnaire (RMDQ)), inappropriate fears and beliefs about lower back pain (the fear, avoidance, belief questionnaire (FABQ)), general quality of life (the Euroqol Group 5 dimension, 5 level questionnaire (EQ-5D-5 L)), Patient Acceptable Symptom State (PASS), consumption of analgesic drugs and nonsteroidal anti-inflammatory drugs (NSAIDs), and overall state of health. Health resource use and days of sick leave (and subsequently the associated costs) will also be recorded. The primary outcome is the presence/absence of a clinically relevant change (improvement of at least 30%) in the VAS score for pain at 6 months.

**Discussion:**

Despite the fact that previous, rather dated recommendations encourage spa therapy for the treatment of low back pain, the current literary corpus is methodologically poor. This protocol has been designed to provide results spanning a thorough range of outcomes at the highest evidence level possible.

**Trial registration:**

ClinicalTrials.gov: NCT03910023. Registered on 10 April 2019.

## Background

Chronic low back pain is a common complaint. It is estimated that about 80% of people will experience lumbar pain at some point in their lives [1]. Epidemiological studies show an increase in the prevalence of chronic low back pain (from 3.9% in 1992 to 10.2% in 2006 in the American population [2]). In a study conducted in 2010, it was estimated that the frequency of low back pain was in sixth place out of the 271 conditions studied, with a prevalence of 9.4% and it was the first cause of disability in the world with 58.2 million disability-adjusted life years (DALYs: number of years lived with a disability) [3].

As concerns treatments, the medications most often prescribed for chronic low back pain pose various problems. Analgesics, recommended as the first line of therapy in most clinical practice guidelines, have limited efficacy that was even considered negligible and insignificant in a recent review [4]. Nonsteroidal anti-inflammatory drugs (NSAIDs) are associated with poor digestive and renal tolerance during long-term use. Opioids have well-documented short-term efficacy, but their long-term efficacy is uncertain, they are sometimes poorly tolerated by older patients, and their long-term use is complicated by addiction in 24% of cases [5, 6].

Given the variable inefficacy of pharmaceutic treatments, different types of physical therapy are an important part of the therapeutic arsenal for low back pain. There have been several trials of spa therapy as one such alternative, which is now recommended for the treatment of chronic low back pain [7, 8]. The latter publications mainly included three studies by a team from Nancy, France that were implemented in the 1990s [9–11]. A systematic review on the subject has been recently published [12], underlining numerous methodological limitations: a lack of primary outcome, failure to perform an intention-to-treat analysis, or no attempt at evaluator blinding. Additionally, certain studies had a waiting list design that could overestimate the effect of the spa treatment. Nevertheless, review studies conclude that spa therapy as part of a multifactorial treatment regimen can improve pain, function, drug consumption and sometimes quality of life [12, 13]. The best level of evidence, however, is still provided by studies conducted in the 1990s.

**Fig. 1:**
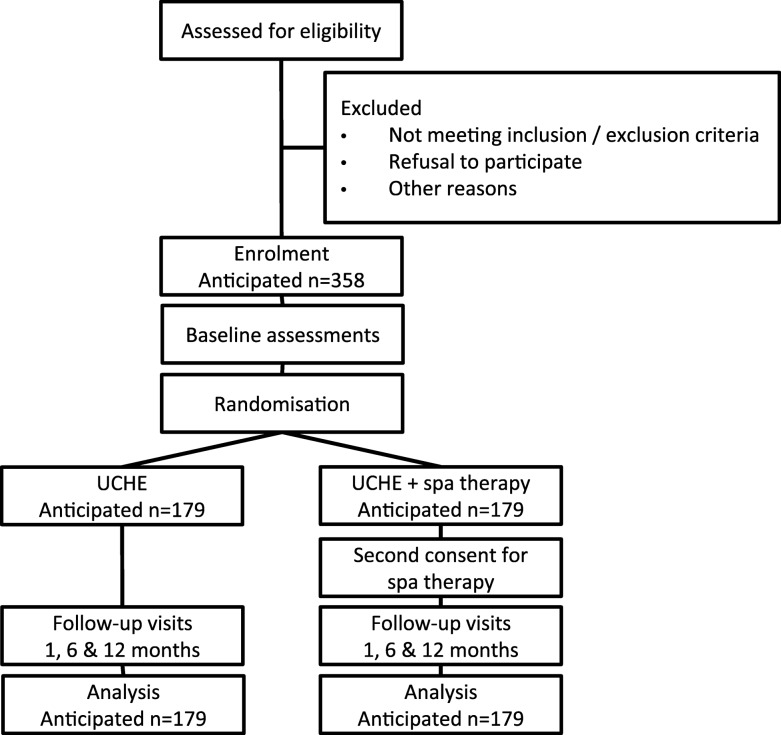
The LOMBATHERM’ trial flow chart. Usual care including home exercise (UCHE) alone will be compared with UCHE plus spa therapy with assessments at 1, 6 and 12 months

We propose to fill this methodological/knowledge gap by implementing a trial with the highest level of evidence possible. Our working hypothesis is that spa therapy in addition to usual care including home exercises (UCHE) will result in greater improvements in pain reduction, associated disability and quality of life in patients with chronic low back pain. It follows that health resource consumption and related costs should also be reduced. The primary objective of this study is therefore to compare the therapeutic effect of UCHE alone versus spa therapy in addition to UCHE for chronic low back pain (Fig. 1). Secondarily, we will also (1) evaluate the therapeutic effects specific to each spa centre, (2) specifically describe effects among subjects who are currently engaged in a professional activity, (3) evaluate treatment tolerance and (4) evaluate per-patient health resource use and associated costs.

## Methods/design

### Trial design

In this prospective, evaluator-blinded, multi-centre, two-parallel-arm, Zelen randomised (1:1) controlled trial, we will compare UCHE alone with spa therapy in addition to UCHE for the treatment of chronic low back pain (Fig. 1).

### Study setting and population

This study will take place in and around well-established spa centres in Europe (see Table 1). Patients living within a 30-km radius of a participating spa centre and meeting eligibility criteria (Table 2) may participate. Recruitment, enrolment and follow-up visits will occur outside of spa centres (and independent thereof) in participating private offices/clinics, public general hospitals and public university hospitals. The single-payer system in France, where most of the participating spa centres are located, offers medical care to all citizens and residents, and the study population should thus represent a wide range of socioeconomic and rural versus urban backgrounds.

**Table 1:** Participating spa centres

Name	Nearby city, Country
Aix-les-Bains	Chambéry, France
Amnéville	Metz, France
Balaruc les Bains	Montpellier, France
Dax	Bayonne, France
Evico Terme	Trentino, Italy
Greoux les Bains	Aix en Provence, France
Hervideros de Cofrentes	Valencia, Spain
Jonzac	Bordeaux, France
Royat	Clermont Ferrand, France
Russian Research Center for Medical Rehabilitation and Balneology	Moscow, Russian Federation
Saint Amand les Eaux	Valenciennes, France
Saint Paul lès Dax	Bayonne, France
Saline Cacica	Northeast Romania
Széchenyi Bath	Budapest, Hungary

**Table 2:** Inclusion and exclusion criteria for participants

Inclusion criteria	Exclusion criteria
Adult patient presenting with chronic low back pain: usual pain of the lumbar region lasting for more than 3 months. This pain may radiate to the buttocks, iliac crest, and does not go past the knee [7]	Patients with specific low back pain
Patient presenting upon inclusion with a current pain intensity on a visual analogue scale (VAS) > or = 40 mm.	Patients with severe depression, psychosis
The patient has signed the informed consent form	Patients who have already had a spa treatment in the previous 6 months
Subject aged over 18 and under 80 (years)	Patients with a contraindication for spa treatment^a^
The patient is a beneficiary of a social security programme (national health insurance)	Patients with a professional activity related to balneotherapy (to avoid any conflict of interest)
	Patients with cruralgia or sciatic pain beyond the knee
	Other treatments that may interact according to the investigator’s judgement
	Patients who live more than 30 km away from the spa^b^

^a^ Immune deficiency, progressive heart disease, progressive neoplasia, infection, chronic bronchitis [14] or foreseeable intolerance to thermal care (intolerance to heat, baths, swimming pool, etc.)

^b^ It will not be convenient to make patients travel more than 60 km per day in their vehicle to deliver between 1 h and 1 h 30 min of outpatient care

In general, eligibility criteria (Table 2) are designed to select for patients with chronic low back pain with a certain level of minimum pain while ruling out cases of specific low back pain. In the case of suspicion of the latter, patients will be aetiologically assessed for rule-out.

### Interventions and study arms

Two interventions are implemented during this trial (Fig. 1): (1) usual care including a home-exercise programme (UCHE) and (2) spa therapy. The UCHE intervention is designed to be a comparator arm that represents standard care, while limiting care heterogeneity. Patients assigned to the control arm of the study will have the UCHE intervention only, and those assigned to the experimental arm will have both the UCHE intervention and the spa therapy intervention.

The UCHE intervention will correspond to current practice (including the continuation of previous treatments, if applicable) as decided by the patient’s general practitioner. They will also be provided with a practical guide on how to manage back pain (the “back book”), including a recommended home exercise programme [15]. The practical guide on back pain and the exercise programme [15] will be presented to the patient by the recruiting investigators during the enrolment visit and prior to randomisation. Use of the “back book” and daily home exercises will be requested of patients and encouraged throughout the study (Table 3); home exercise observance will be queried at each follow-up visit (Table 3) by asking the patient on what days during the previous week he/she performed the required exercise routine. The recruiting investigator will further encourage and insist that all study participants are to keep trial participation a secret from evaluating investigators.

**Table 3:** The LOMBATHERM’ protocol schedule for enrolment, interventions, assessments, and visits for participants

Visit/event:	Enrolment visit^a^		Intervention period	Follow-up visits^b^		
	Baseline	Allocation		1	2	3
Target chronology (days, weeks, months)	D0	D0 (after baseline)	D1–D60	M1^c^ (D28–D60)	M6 ± 4 W	M12 ± 4 W
**Enrolment and allocation**						
First study presentation and consent 1	✓					
Randomisation		✓				
For those patients randonmised to the experimental arm, presentation of additional spa-treatment and consent 2		✓				
**Interventions**						
Explanation about low back pain and home exercise training for all patients	✓					
Daily exercises at home using the “back book” for all patients^d^			*Throughout intervention and follow-up periods*			
Eighteen consecutive days of spa-therapy (except Sundays) for patients in the experimental arm			✓			
**Observance**						
Home exercise observance				✓	✓	✓
Spa therapy observance (experimental arm only)			✓			
**Harms**						
Tolerance: adverse event characterisation and reporting			*Throughout intervention and follow-up periods*			
**Assessments**						
Demographics and disease history^e^	✓					
Visual analogue scale for low back pain	✓			✓	✓	✓
The Rolland and Morris (RMDQ) scale	✓			✓	✓	✓
The FABQ	✓			✓	✓	✓
The EQ-5D-5L questionnaire	✓			✓	✓	✓
Patient Acceptable Symptom State	✓			✓	✓	✓
Global opinions on state of health^f^	✓			✓	✓	✓
Drug consumption in the past 72 h	✓			✓	✓	✓
Health resource use since last visit^g^				✓	✓	✓
Cost of care^h^						✓
**Blinding success**						
Guess-the-group question for evaluators						✓

*D* day, *M* month, *W* week, *FABQ* Fear, Avoidance, Belief Questionnaire, *EQ-5D-5L* Euroqol Group 5 dimension, 5 level questionnaire

^a^Enrolment visits are performed by recruiting investigators, who are not the same as evaluating investigators

^b^Follow-up visits are performed by evaluating investigators, who are not the same as recruiting investigators

^c^Or just after the end of spa therapy, if applicable

^d^The patient will also be provided with a practical guide on how to manage back pain (the “back book”), including a recommended home exercise programme [15]

^e^Age, sex, duration of current episode of low back pain, signs of spread of osteoarthritis, pain spreading, history of lumbar surgery, need for frequent position changes, bad mood and irritability, sleep disorders due to back pain

^f^Semi quantitative scales that reflect (1) the patient’s and, separately, (2) the physician’s overall opinion concerning the patient’s state of health

^g^Resource use including infiltrations, hospitalizations, physical treatments (massages, traction, physical therapy) and medical imaging of the lumber spine will be tracked

^h^Costs will be estimated using resource-use data gathered during visits and the French National Cost Scale. Indemnities related to days of sick leave will be included in cost estimates

The composition of the spa therapy intervention is based on previous results in the domain. Massage [16–21], heat therapy [20] and water exercises [22] have all been demonstrated as helpful for low back pain. Water exercises in particular have similar efficacy to regular exercise regimens, and are sometimes better tolerated [22]. For the present study, the spa therapy intervention consists of 18 consecutive days of spa therapy (3 weeks of treatment, excepting Sundays), that must occur within 60 days of randomisation (Table 3). The latter will always combine mineral water with physical and hydrotherapeutic treatments, including massages, heat and water exercises: (1) pool activities will be carried out by qualified and trained physiotherapists. Small patient groups (limited to 8–15 patients at a time) will participate in pool sessions with 10 min of free bathing and 15 min of supervised exercises in water at 35 °C; (2) underwater massages on painful areas will be performed by qualified and trained physiotherapists in 10-min sessions with water jets at a temperature of 38 °C; (3) mud applications at a temperature of 45 °C will be performed in 15-min sessions, and will include all painful areas (even outside the lumbar region if necessary). In addition to the latter three criteria, further treatments may be added by consensus of spa practitioners in each spa centre. Patients randomised to the spa therapy arm will be invited by the recruiting investigator to keep trial participation and spa therapy a secret from evaluating investigators. Their participation in spa therapy (observance) will be recorded by spa centre staff.

If required, treatment-stopping for home-exercises will be decided via collaboration between the patient and his/her evaluating doctor. Treatment-stopping may occur for spa-therapy in case of an adverse event, is decided on a case-by-case basis by the on-site doctors overseeing spa-therapy, and does not result in study exclusion.

### Allocation and blinding

Following initial consent covering the UCHE intervention and subsequent baseline assessments, patients will be randomly assigned to either control or experimental groups in a 1:1 allocation ratio (Fig. 1, Table 3). In line with a single Zelen design [23, 24], patients randomised to the experimental group will be informed about the additional spa therapy intervention and invited to sign an additional consent form; furthermore, these patients can refuse spa therapy, but statistical analysis will be performed on an intention-to-treat basis. Patients allocated to the control group have already consented to the UCHE intervention and will proceed without information on spa therapy; this design is necessary to avoid bias associated with “resentful demoralisation” effects [24].

Allocation is performed using a computer-generated randomisation schedule and stratified by the recruiting investigator/spa (the latter are paired, but separate) and by whether or not the patient has a professional activity. Baseline evaluations and the subsequent randomisation are performed by the recruiting investigators through specific modules administered via a password-protected Internet-accessible electronic case report form (eCRF). The randomisation module will become accessible to investigators only after key elements of baseline data have been entered in the eCRF.

To blind the study as much as possible, follow-up assessments are performed during specific follow-up visits (see Table 3) by an “evaluating” physician, who is different from the “recruiting” investigator. Every attempt will be made to keep evaluating investigators blinded. Specifically, study participants will be instructed to not mention study participation or spa therapy to their evaluating physicians (at the end of the final follow-up visit, the study clinical research associate (CRA) will ask the evaluator a guess-the-group question in order to evaluate the success of blinding). As concerns follow-up care, emergency situations requiring specific unblinding procedures are not foreseen in this protocol. Finally, the statisticians will be blinded, i.e. study arms will be referred to as “A” or “B” when the database is presented for analysis. Unveiling of the study arms will occur after completion of planned statistical analyses.

### Primary outcome

The primary outcome is the presence/absence of a clinically relevant change (improvement of at least 30%) in the visual analogue scale (VAS) score for pain at 6 months after baseline assessments (see Table 4). This choice is justified by the fact that it is relevant to the patient, figures in international recommendations [35–37], and is qualitative in nature. A qualitative outcome was also preferred because it should minimise the role of placebo effects, which are often significant for quantitative pain variables but not so for qualitative ones [38], and minimise effects associated with a lack of evaluator blinding, as is the case for self-evaluation questionnaires [39]. For the purposes of this study, a clinically relevant change in VAS scores for pain is defined by an improvement of at least 30% [40, 41]; improvements below this value will be considered as treatment failures (absence of clinically relevant change). Cases where patients demonstrate a 30% improvement in VAS scores for pain but nevertheless require hospitalization for back pain during the study follow-up period will also be considered as treatment failures.

**Table 4:** Outcome measures

Patient-specific measure	Analysis metric and time frame	Planned analysis type
Number of patients with a clinically relevant change in visual analogue scale (VAS) score for pain, defined by an improvement of at least 30%; cases where patients demonstrate a 30% improvement in VAS scores for pain but nevertheless require hospitalization for back pain during the study follow-up period will be considered as treatment failures (absence of clinically relevant change)^a^	Proportion of patients with clinically relevant change for pain between baseline and 6 months	Comparison of distributions^b^
Huskinsson’s VAS for pain [25]. As recommended by national French health authority standards [26], this will address pain over the last 8 days	Repeated measures at baseline and 1, 6 and 12 months^b^	Mixed model for longitudinal data^c^
The Rolland & Morris (RMDQ) pain scale [27]: in this validated French version [28], a 24-question scale (with scores ranging from 0 (no impact) to 24 (maximum impact)) quantifies the impact of low back pain on daily life		
The Fear Avoidance and Belief Questionnaire (FABQ) assesses inappropriate fears and beliefs concerning back pain; the validated French version will be used [29]		
The EQ-5D-5L questionnaire [30, 31] will be used to track changes in quality of life. This self-administered questionnaire consists of two pages: the first contains the EQ-5D descriptive system and the second a visual analogue scale. The descriptive system has 5 dimensions (mobility, self-care, usual activities, pain/discomfort, anxiety/depression), each described by 5 levels of intensity (“no problems”, “slight problems”, “moderate problems”, “severe problems” and “extreme problems or complete inability”). The respondent must indicate one intensity level for each dimension	Repeated measures at baseline and 1, 6 and 12 months Proportion of patients with clinically relevant changes according to van der Roer et al. [32] at 1, 6 and 12 months	Mixed model for longitudinal data^c^ Comparison of distributions^b^
Patient Acceptable Symptom State (PASS): yes/no response to the following question: “If you take into account all the activities you have in your daily life, the importance of your pain and your disability, do you consider your condition as satisfactory?” [33]	Proportion of patients with patient-acceptable symptoms at baseline and 1, 6 and 12 months	Comparison of distributions^b^
Overall opinion of the patient on his/her state of health (5-point Likert scale)	Proportion of patients in each state at baseline and 1, 6 and 12 months	Comparison of distributions^b^
Overall opinion of the evaluating physician on the patient’s state of health (5-point Likert scale)		
Daily drug consumption over the past 72 h: • Analgesics (in milligrammes of paracetamol and morphine equivalents) • NSAIDs (reported in milligrammes and as a percentage of the maximum dose) [34] • Corticosteroids (in milligrammes equivalent prednisone) • Benzodiazepine muscle relaxants: % of maximum dose	Repeated measures at baseline and 1, 6 and 12 months	Mixed model for longitudinal data^c^
Number of infiltrations (epidural, posterior articular)	Centrality for the cumulative number of infiltrations at 12 months	Comparison of centrality^d^
Estimated per-patient cost for 1 year of care (based on observations of health resource use throughout the study: drug consumption, hospitalizations, imaging)	Centrality for the cumulative cost of care at 12 months	Comparison of centrality^d^

^a^ The primary outcome

^b^ The comparison of the number of patients improved (or other proportion) will be performed via distribution comparison tests for independent groups (either the uncorrected χ^2^ test, or Fisher’s exact test if the conditions for the χ^2^ test are not met)

^c^ A mixed model with patients as a random effect and study arm as a fixed effect will be used. A significant time × group interaction would indicate differences in the speed of recovery/change between groups

^d^ Means will be compared using the *t* test for independent groups when variable distributions are normal according to the Shapiro-Wilks test, or otherwise via the Mann Whitney test for comparing medians

### Secondary outcomes

Further outcomes are presented in Table 4 and cover changes over time in VAS pain scores, and changes over time assessed by three validated questionnaires: (1) the impact of lower back pain on daily life (the Rolland and Morris Disability Questionnaire (RMDQ) [27, 28]), (2) inappropriate fears and beliefs about lower back pain (the fear, avoidance, belief questionnaire (FABQ) [29]) and (3) a general measure of quality of life (the Euroqol Group 5 dimension, 5 level questionnaire (EQ-5D-5 L questionnaire) [30, 31]). Patients will also be questioned about how they perceive the acceptability of their symptoms, using the Patient Acceptable Symptom State (PASS) [33]. The overall opinion of the patient and the physician on the patient’s state of health (5-point Likert scale) will be recorded. Finally, per-patient health resource use will be quantified by estimating drug consumption, infiltrations, hospitalizations, imaging and sick leave. The latter will be used, in conjunction with the French national cost scale, to estimate the cost of care after 12 months of follow up for each patient (Table 4).

### Sample size

Data from two unpublished, open-label studies on typical patients receiving spa therapy indicate estimated rates of clinically relevant change in VAS pain scores of 47% (8/17) and 38% (10/26). Our hypothesis is that the proportion of patients experiencing improvement at 6 months will be 40% in the experimental arm (UCHE plus spa treatment) versus 25% in the control arm (UCHE only). Considering a type 1 risk level set at 4% (1% will be used for an interim analysis following the inclusion of the first 100 patients) and a type 2 risk level set at 20% (i.e. statistical power of 80%), the minimum sample size required is 322 participants. To maintain power for analysis on observed data and expecting a drop-out rate of approximately 10%, this estimate is increased to a total of 358 paricipants (179 per study arm).

### Recruitment and visits

Recruitment will be assisted by advertising (not mentioning spa therapy) in the local press and by posting information in local pharmacies, physicians’ offices or clinics. In the case of recruitment difficulties, local companies known to have high rates of workers on sick leave for low back pain may be contacted. Following the advertising campaign, telephone contact between responding, potential study participants and a clinical research technician (CRT) will result in referral to a recruiting investigator (depending on location), who is responsible for obtaining consent and for enrolment, baseline evaluations and randomisation. The enrolment visit will start with an initial presentation and provision of informed consent for the UCHE intervention (Table 3). Following presentation of the “back book” and baseline evaluations, participants will be randomised and those allocated to the experimental arm will be invited to sign an additional consent form for spa therapy. As no biological collections or ancillary studies are foreseen in this protocol, further consent procedures beyond the latter are not required. Appointments will be made for spa therapy (in the next 60 days (maximum) for patients consenting to spa therapy in the experimental arm) and for follow-up visits with evaluating physicians for all patients in both arms at months 1, 6 and 12. The specific assessments required at baseline and during follow-up visits are detailed in Table 3 and closely follow the study outcomes plus the following: demographic data and disease history at baseline, home exercise observance and any required harms reporting. No specific post-trial care (beyond routine care) is foreseen.

### Promoting participant retention and completeness of follow up

Continued contact between study CRTs and patients is encouraged. Patients are requested to contact the study CRT in the case of visit unavailability or other problems. Conversely, the study CRTs will be contacting patients during the week preceding follow-up visits to provide a visit reminder and/or rescheduling as required.

### Harms

Few prospective studies have sought to evaluate the adverse effects and complications that may occur during a spa treatment [42–45]. Due to the absence of control groups, imputability is often not established and few studies clearly describe side effects. Among patients undergoing spa therapy, with or without radon, Franke et al. [39] observed: an increase in pain (7/1), hypertension (2/1), fatigue (2/1) or coloration of the skin and nails (2/0). Gáti et al. [46] observed three cases of respiratory tract infection, one case of cardiac arrhythmia, hypertension and cardiac decompensation, one case of cystitis and one case of gastroenteritis in relation to spa therapy. Another study reports a complete absence of adverse effects [37]. In other publications, adverse effects are not discussed. Based on adverse event reports from the largest studies [47, 48], no serious adverse events are expected. Based on the adverse effects observed during spa treatment for knee osteoarthritis, and in epidemiological studies, we expect to observe benign infections of the upper airways, increased pain or potentially leg erysipelas. A rarer but theoretically possible effect is the occurrence of opportunistic pulmonary infections (legionella) - a few cases and case series were described in the 1980s. These infections have become rare following a change in water disinfection methods in the spa centers [49].

Additional events that may be expected because they are associated with physical exercise in general are respiratory disorders and shortness of breath, cardiovascular disorders (chest pain, myocardial infarction, cardiac arrhythmia, tachycardia, vagal discomfort, hypotension or hypertension, fatigue and injuries (cramps, muscle aches, sprains, strains, tears, fractures)).

Harms reporting will be carried out in compliance with the French regulations in force (or their equivalent in other countries as appropriate) throughout the study (see Table 3). There is no anticipated harm or compensation for trial participation.

### Statistics

Statistical analyses will be performed by the Department of Medical Information at the University Hospitals of Montpellier, Montpellier, France, using the R programming environment [50] or SAS (enterprise guide V.7.1 or higher). An intention-to-treat structure will be implemented. The intention-to-treat population will include all randomised patients and will respect their allocations, regardless of whether or not they started the allocated interventions. Analyses may be adjusted according to clinical or inclusion criteria to take into account population heterogeneity. Though generally presented here, a more detailed statistical analysis plan will be drafted prior to the end of participant inclusion (and made available at https://osf.io/ahpwy/).

Descriptive statistics (for the total population and for each arm) will be provided as means with standard deviations for quantitative variables with normal distributions according to the Shapiro-Wilks test, or as medians with interquartile variables for other quantitative variables. Numbers and percentages will be provided for categorical variables.

As indicated in Table 4, the primary analysis is the comparison of the proportion of patients with a clinically relevant change in pain at 6 months; this will involve the uncorrected chi square (χ^2^) test for independent groups, or alternatively Fisher’s exact test if the conditions for the χ^2^ test are not met. We expect to perform an interim analysis of the number of patients in both groups with clinically relevant improvement as soon as there are 100 patients suitable for analysis with 6 months of follow up. The purpose of the latter is to assess futility, and a difference between arms (intervention – control) < 0 will stop the trial.

As concerns secondary analyses, comparisons of qualitative variables will be performed in the same manner as for the primary analysis. The centrality of quantitative variables will be compared between arms using parametric (*t* test) or non-parametric (Mann Whitney test) analysis for independent groups, as appropriate. For longitudinal data, the preferred method will employ mixed models with patients set as a random effect and study arm as a fixed effect; an appropriate interaction term (group × time) will be used to detect differences between study arms in the rate of change. In addition, a medico-economic analysis comparing the two patient management strategies will be conducted according to international [51] and French recommendations [52]. A cost-utility analysis will be carried out, with utility values evaluated using the EQ-5D-5 L. The following direct medical costs will be considered: hospitalizations and consultations, transport, drugs and laboratory assessments.

Variables that are used for stratification (spa or presence of a professional activity) may be used in sub-group analyses. Covariate-interaction tests are the preferred method for detecting sub-group differences [53]. Statistical analyses will be adjusted on variables found to be imbalanced between arms. Sub-group tests will be considered as exploratory in nature.

The general hypothesis of the study supposes more improvement in the experimental arm as compared to the control arm. A difference will be considered statistically significant when the type I error rate is less than or equal to 0.05. As concerns missing data, patients lost to follow up will not be considered as withdrawn from the study but will be analysed as failures for the primary endpoint. Any deviations from the statistical analysis plan must be authorized by the study methodologist and will be thoroughly documented and justified in the study analysis report.

### Data entry and quality verifications

Individual patient data will be entered in a password-protected, web-accessible eCRF (Ennov Clinical; https://en.ennov.com) by participating investigators or their approved delegates. A paper version of the eCRF will be made available at https://osf.io/ahpwy/, and can be used to facilitate field requirements for speed or as back up in the case of electronic system unavailability. Data entry in the eCRF will be performed in real time as much as possible in order to take advantage of specifically designed data format, range and coherence rules. The Clinical Research and Epidemiology Unit at the University Hospitals of Montpellier (Montpellier, France) is responsible for randomisation lists, database maintenance and data management procedures. Data in the eCRF will be audited against resource documents by data-monitoring personnel throughout the study, and corrections made via a traceable system of queries and responses.

### De-identifying and using data

In the eCRF (and paper forms if required) and subsequent study database, participants will be de-identified and only the following will be used for identification purposes during the study: two initials, year of birth and study number. Any publicly available deliverables will be completely de-identified (individual spa centres and patients are referred to by numbers only when per-patient differentiation is required, and absence of any information that may be used to re-identify any individual).

As explained in the patient information materials (https://osf.io/ahpwy/), the French Public Health Code allows LOMBATHERM’ study data, including the data of patients who withdraw their consent or who are lost to follow up, to be used specifically for the purposes of this study, and only this study, unless the patient so opposes. Re-use of the data by other teams via data sharing is subject to approval by the Commission nationale de l’informatique et des libertés (National Commission for Data Protection) (France), as stated in the data sharing plan (https://osf.io/ahpwy/).

### Monitoring trial conduct

The project will be monitored by clinical research associates delegated by the Sponsor (Sponsor CRAs) via a subcontract with the University Hospitals of Montpellier. Monitoring will be performed by regular on-site or remote inspections at investigating centres (enrolment visit, follow up according to the pace of inclusions and a closure visit). All monitoring visits will be the subject of a written report and will cover consent procedures and protocol adherence.

### Study steering committee and communication activities

The LOMBATHERM’ steering committee (RF, NM, CMS) will (1) supervise study implementation and execution, (2) determine study continuation following interim analysis and (3) oversee communication activities in accordance with the data sharing plan. Key study documents (participant information materials, statistical analysis plan, analytic code, data sharing plan) will be made available to the public on the study’s Open Science Framework website: https://osf.io/ahpwy/. The final study database will be available to all study investigators immediately after the database is locked. The final study report will be communicated to the study Sponsor and to French authorities within 12 months of database freezing. Aggregated trial results will be made available to the public on ClinicalTrials.gov and by publication in a peer-reviewed journal. At this time, the use of professional writers who are not recognized authors is not foreseen, and authorship will be attributed by general consensus and according to the criteria stipulated by the International Committee of Medical Journal Editors (http://www.icmje.org). In addition, results will be made available to any study participant upon request, as per French regulations in force. Finally, the study data sharing plan stipulates that requests to the AFRETh (Association Française pour la Recherche Thermale, 1 rue Cels 75,014 Paris, France; http://www.afreth.org) for individual datasets can be made anytime following full publication of results.

## Discussion

The LOMBATHERM’ protocol aims to document a thorough range of endpoints highly relevant to the targeted population of patients with low back pain, including changes in pain and symptom acceptability, associated disability, fears and beliefs and quality of life. Should the expected positive effects of spa therapy in addition to usual care be confirmed, a safe, novel physical therapy regimen will be added to the treatment arsenal available for these patients who are difficult to treat.

Care was taken to reduce sources of bias in as much as possible by selecting a clinically relevant primary endpoint and implementing a Zelen randomisation procedure (which eliminates “resentful demoralisation” effects [24]). Further advantages of the Zelen design are an improvement in patient blinding for the control arm, making treatment discussion with the patient more straightforward and closer to “routine” clinical practice, potentially increasing recruitment rates and decreasing post-randomisation withdrawal. However, a single-Zelen design also has a certain limitation that must be taken into account, i.e. the possibility of patient crossover between arms. The latter may dilute the treatment effect within an intention-to-treat analysis, thus providing an underestimated or conservative estimate [24].

Further limitations to the study include a lack of an independent data and safety monitoring board, which was deemed unnecessary due to the low-risk status of this trial. One must also consider the single-blind nature of the protocol, which is understandable given the impossibility of implementing full blinding of the patients receiving spa therapy. Furthermore, there is no way to guarantee the blinding of evaluators, which highly depends on patient collaboration. Nevertheless, the partial blinding of patients (control arm via the Zelen randomisation), the assessing physician, therapists and statisticians have all been addressed. The success of evaluator blinding will therefore be assessed by a guess-the-group question at the end of the final follow-up assessment.

Chronic low back pain has a strong impact on patients’ daily lives and entails significant costs in terms of social protection/insurance [54]. It frequently affects subjects during periods when they are the most productive during their professional careers [55]. A French study estimated the direct costs of low back pain in France to be 1.6 billion euros in 2002 [56]. Half was attributable to hospital expenditure (800 million euros). Low back pain required 13 million consultations and drug costs amounting to 570 million euros. These expenditures increased by 156% compared to 1993, due to an increase in the number of patients treated (+ 54%) and the cost per patient (+ 2.5% per year). In addition to health resource usage, the LOMBATHERM’ protocol will also collect data on the days of sick leave for back pain required by participants. This will allow a first estimate of recent changes in direct and indirect costs associated with spa therapy.

An additional interesting aspect is the follow-up period extended to 12 months. Classically, 90% of lumbar pain episodes are considered as resolved in the following month, but reality is more complex. An observational study of 490 patients consulting for a lumbar episode showed that 59% were satisfied with only one consultation and 32% had two consultations in 3 months. On the other hand, only 21% at 3 months and 25% at 1 year have complete recovery of their functional capacity and resolution of their pain [57]. The repeated follow-up visits stipulated by the present protocol will provide a more complete vision of the durability of therapeutic efficacy as related to spa treatment, as well as the different health resource pathways taken by patients.

In conclusion, the LOMBATHERM’ trial has been designed to provide results spanning a thorough range of outcomes at the highest evidence level possible. Low back pain is the most common symptom in patients undergoing spa therapy: 70% of the population aged 54 to 65 years who attend health examination centers have experienced low back pain and of these, 84% of the sub-population that went to spas [58]. It should be noted that very few studies are conducted in this age group, which is generally not considered in recommendations. The proposed study will therefore fill an important knowledge gap in the domain.

## Trial status

Protocol version 2.1 (18 October 2019). The first patient was included on 15 June 2019. Recruitment is currently ongoing. The a priori end of recruitment is planned for 15 December 2021, but may be re-adjusted by protocol modification with ethics committee approval.

## Data Availability

Data sharing is not applicable to this article as no datasets were generated or analysed during the current study.

## Abbreviations

AFRETh: Association Française pour la Recherche Thermale, 1 rue Cels 75,014 Paris, France; http://www.afreth.org
CRA: Clinical research associate
CRT: Clinical research technician
DALY: Disability-adjusted life year
eCRF: Electronic case report form
EQ-5D-5L: Euroqol Group 5 dimension, 5 level questionnaire (https://euroqol.org/eq-5d-instruments/eq-5d-5l-about/)
FABQ: Fear, Avoidance, Belief Questionnaire
NSAID: Nonsteroidal anti-inflammatory drug
PASS: Patient Acceptable Symptom State
RMDQ: Rolland and Morris Disability Questionnaire
UCHE: Usual care including home exercise
VAS: Visual analogue scale
